# Oral and Dental Considerations in Management of Sickle Cell Anemia

**DOI:** 10.5005/jp-journals-10005-1301

**Published:** 2015-08-11

**Authors:** Sonu Acharya

**Affiliations:** Reader, Department of Pedodontics and Preventive Dentistry, Institute of Dental Sciences, Bhubaneswar, Odisha, India

**Keywords:** Dental management, Oral considerations, Sickle cell anemia, Sickling.

## Abstract

Sickle cell anemia is a genetic disease that primarily affects the black population. This anemia is due to a homozygous state of the abnormal hemoglobin S. An alteration occurs on the DNA molecule involving the substitution of the amino acid valine for glutamic acid at the sixth position on the beta polypeptide chain. This biochemical variation on the DNA molecule creates a physiological change that causes sickle-shaped red blood cells to be produced. The sickle-shaped cells are the result of the hemoglobin S being deoxygenated. This case report presents a case of 16-year-old female with sickle cell disease and its dental management.

**How to cite this article:** Acharya S. Oral and Dental Considerations in Management of Sickle Cell Anemia. Int J Clin Pediatr Dent 2015;8(2):141-144.

## INTRODUCTION

Sickle cell anemia is a genetic disease caused by replacement of glutamic acid by valine in position 6 at the N-terminus of the beta-chain of globin, thus resulting in hemoglobin S.^1^ Under conditions of hypoxia, erythrocytes that predominantly contain hemoglobin S take on a shape resembling a sickle.^2^ This sickling is reversible through increased oxygen levels, although constant changes in shape result in cell membrane lesions that make the cells rigid, preventing them from returning to their normal state. The reduction in oxygen-transport capacity results in circulatory difficulties, including vaso-occlusive conditions, which diminishes the lifespan of the red blood cells to approximately 20 days. Sickle cell disease (SCD) usually manifests early in childhood. For the first 6 months of life, infants are protected largely by elevated levels of Hb F; soon thereafter, the condition becomes evident.^3^ The most common oral manifestations of sickle cell disease are mucosal pallor, yellow tissue coloration, radiographic abnormalities, delayed tooth eruption, disorders of enamel and dentin mineralization, changes to the superficial cells of the tongue, malocclusion, hyperce-mentosis, and a degree of periodontitis that is unusual in children.^4^ For dental treatment to be carried out, it is recommended that dental surgeons have an understanding of the pathophysiology of this disease, enabling them to determine treatment plans so that they can also take systemic conditions into consideration.

## CASE REPORT

A 16-year-old girl visited, accompanied by guardian, to the department of pediatric and preventive dentistry with the complain of multiple dental decay. On taking a medical history guardian revealed the patient was a known case of sickle cell disease and has been under medication. She had been hospitalized thrice and had been given blood transfusion four times since being detected with sickle cell disease. Blood investigations revealed the blood picture characteristic of sickle cell anemia.

**Fig. 1:**
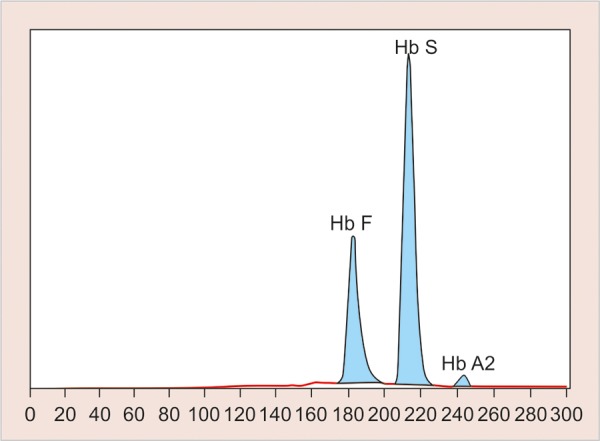
Hemoglobin electrophoresis showing HbF and HbS

**Fig. 2:**
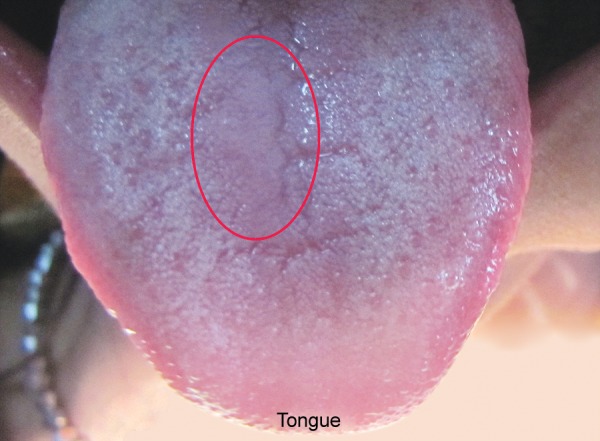
Smooth tongue of sickle cell anemia

**Fig. 3:**
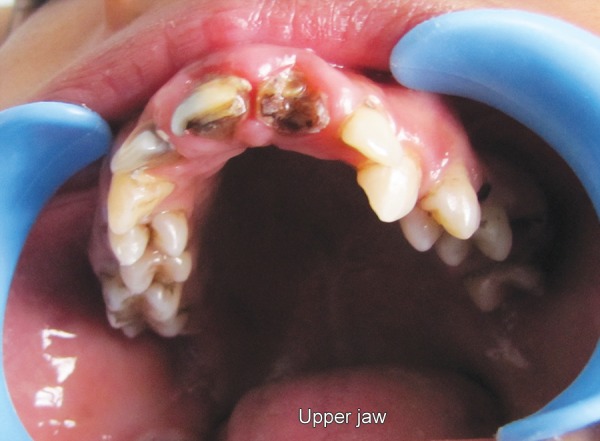
High arched palate and caries in maxillary teeth

**Fig. 4:**
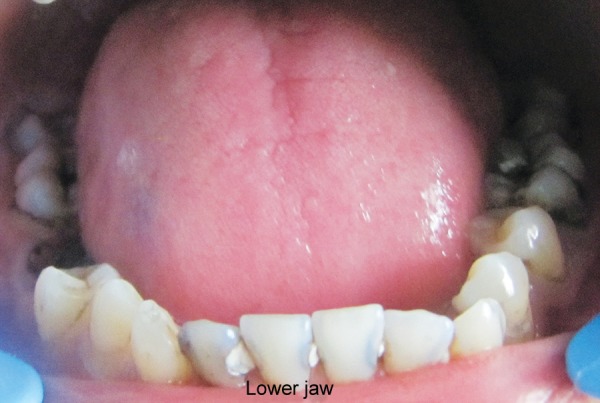
Multiple caries in mandibular teeth orthopantomograph

**Fig. 5:**
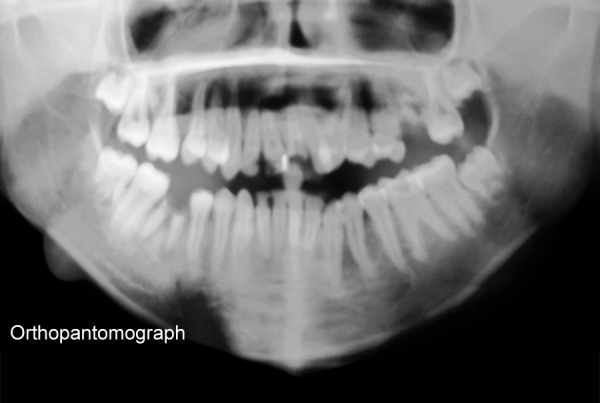
Orthopantomograph showing massive resorption in lower border of mandible

**Fig. 6:**
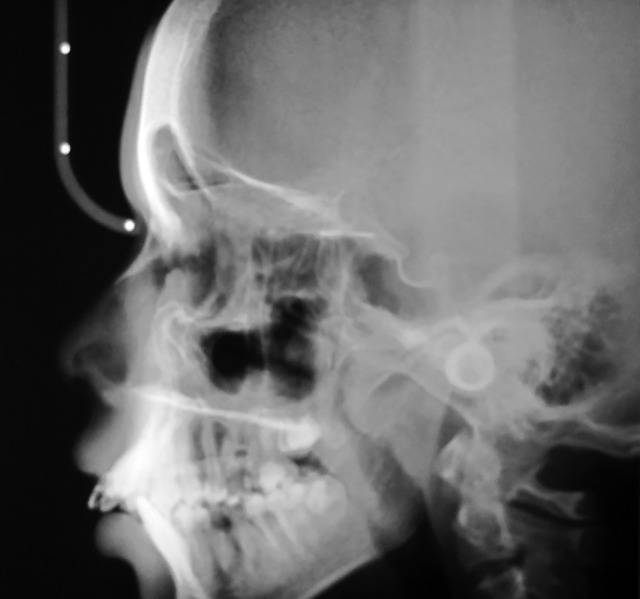
Lateral skull view showing hair on end appearance

**Fig. 7:**
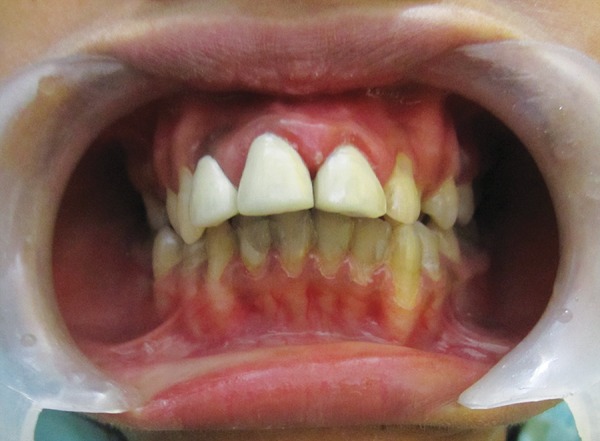
After rehabilitation

Hemoglobin electrophoresis revealed HbS and HbF (Fig. 1). At present visit, the condition was under control because of blood transfusion and chemotherapy. On examination, the patient was conscious, cooperative with normal gait and was well-oriented. Patient also complained of joint pains which is again a sign of sickle cell disease. Extraoral findings were not so suggestive. Evaluation of the oral soft tissues showed signs suggestive of sickle cell anemia, including mucosal pallor and abnormalities of tongue morphology known as ‘smooth tongue’ (Fig. 2). Oral examination (Figs 3 and 4) after dental prophylaxis also revealed multiple active carious lesions in 16 15 25 26 36 31 32 41 42 with abscess in relation to mandibular right second molar (47). The patient had carious broken 11 and 21. Further the child had root stump of 26. The jaw relation was Angle class 1 with increased overjet and incompetent lips. The orthopantomograph radiographic (Fig. 5) findings of distinct radiopaque areas caused by repairs to bone infarction were present in both the maxilla and the mandible. The radiographs also revealed loss of the normal trabecular pattern with increased radiolucency due to decreased number of trabeculae and increased medullary spaces secondary to compensatory hyperplasia; resorption of the lower edge of the mandible on right side. There was a coarse trabecular pattern of ‘staircase’ shape (present mainly in the interproximal bone because of trabeculae that formed horizontal rows), presence of projections similar to ‘hair strands’ due to secondary formation of bone tissue as compensation for resorption that occurred during bone marrow expansion. The lateral skull radiograph (Fig. 6) revealed thickened diploe; the trabeculae are coarse and are running perpendicular to the inner and outer table giving a characteristic appearance of ‘hair-on-end pattern’. After carrying out the physical and complementary examinations it was decided for a complete oral rehabilitation of the child after consultation with a pediatrician as the child had multiple foci of infection which could lead to a sickle crisis. Composite restorations were planned and done for 31 32 41 42 36 15 16 25. Endodontic procedures were performed for 21 22 11 with post and core for 11 and 21 and porcelain fused to metal crowns on them (Fig. 7). Extractions for 47 and 26 were done and rehabilitation planned for missing teeth. All the procedures were carried under antibiotic coverage and strict aseptic conditions. To relieve pain in jaws, advise for over-the-counter pain relievers like NSAIDs was given and if pain is severe more potent analgesics like morphine can be given. It was also advised to the patient that in case of severe painful crises hospitaliza-tion is a better option. Adjuvants like antidepressants, antihistamines can also be given. These are heterogenous compounds which potentiates the analgesic effects of analgesics and also ameliorates their side effects. After this initial therapy, the patient and her family were advised about future oral healthcare. They were also given dietary advice to promote a non-cariogenic meal plan, and received further instruction in tooth-brushing, the use of dental floss and tongue cleaning. Return visits at 3 to 4 weekly intervals for fluoride applications and oral hygiene checks were arranged.

## DISCUSSION

Sickle cell anemia is associated with abnormal hemoglobin and the genes that make it. Our genes are encoded with two chromosomes to make hemoglobin. People who have inherited sickle cell anemia have two copies of the sickle cell gene. They inherit the sickle cell gene from each parent; hence, both parents must have the sickle cell trait or gene to pass on to their children. Normal red blood cells are smooth and round and have easy access through blood vessels to carry oxygen to all parts of the body. In SCA, the abnormal hemoglobin sticks together when it gives up its oxygen to the tissues. Sickle-shaped cells do not move easily through blood. They are stiff, sticky and ‘C’ shaped and is susceptible to forming clumps. The clumps of sickle cells block blood flow in the blood vessels that lead to the limbs and organs. In general, the clinical manifestations of the disease are first noted between 6 months and 3 years of age. The reason for the delay in the appearance of symptoms is due to the protective effect of the fetal hemoglobin (HbF).^5^ At birth, 50 to 95% of the hemoglobin is HbF. It gradually declines 2 to 4% each week postpartum and is replaced by HbS. The protective effect of the HbF is lost and symptoms of the disease then develop. In the sickle cell trait, only 20 to 45% of the hemoglobin is HbS and the rest is normal hemoglobin. Moreover, only one of the β chains is thought to be abnormal, where both β chains are abnormal in SCD. Microvascular occlusions are thought to be responsible for most of the clinical manifestations of SCD.^6^ They may involve most of the organ systems of the body and are frequently characterized by pain as well as by various signs and symptoms of organ dysfunction. The most characteristic manifestations of SCD is the sickle cell crisis. It consists of an episode of severe pain of 1 to 2 weeks duration, often accompanied by a low-grade fever and leukocytosis.^7^ Our case also reported severe joint pain and fever few days prior to her visit. A crisis may be precipitated by infection, dehydration or acidosis or may be associated with any identified etiology. Hematological manifestations are characterized by anemia with hemoglobin values of 5 to 9 g/dl. Red blood cell survival time is markedly decreased. Normoblastic hyperplasia in the marrow, aids in the maintenance of constant hemoglobin levels. Reticulocyte count usually ranges from 5 to 25%. The white blood cell count is generally in the range of 10,000 to 20,000 cells/mm^3^ with the platelet count being normal to slightly elevated. Hemoglobin electrophoresis with the separation of HbS, a positive HbS solubility test and a positive sickle preparation will accompany the preceding hematological findings.^8^ The initial evaluation of the patient with a sickle hemoglobinopathy must include a thorough history and physical examination. In most instances, the patients with classic SCD are aware of their diagnosis and have experienced multiple hospitalizations for painful crises, episodes of severe anemia, multiple blood transfusions and recurrent bouts of bacterial pulmonary infections.^9^ The case mentioned in our report also had been hospitalized thrice following sickle crisis and seizures. A medical consultation with the patient’s physician must be on file with verification that the condition is stable enough for treatment. All appointments should be short and stress free, avoiding long and complicated procedures.^10^ When treating patients with SCA the dental surgeon should institute aggressive preventive dental care including: oral hygiene instructions, diet control, tooth brushing and flossing instructions, as well as fluoride gel applications.^11^ Although the oral manifestations are not exclusive to this disease, they may suggest a diagnosis of sickle cell disease.^12^ In this report, the mucosal pallor was very evident and observed primarily in the oral and labial mucosa. The smooth appearance of the tongue was not compounded by poor hygiene of this organ because of the absence of a tongue coating. The radiographic findings of distinct radiopaque areas caused by repairs to bone infarction were present in both the maxilla and the mandible. The radiographs also revealed loss of the normal trabecular pattern with increased radiolucency due to decreased number of trabeculae and increased medullary spaces secondary to compensatory hyperpla-sia; thinning of the lower edge of the mandible. There was a coarse trabecular pattern of ‘staircase’ shape (present mainly in the interproximal bone because of trabeculae that formed horizontal rows), presence of projections similar to ‘hair strands’ due to secondary formation of bone tissue as compensation for resorption that occurred during bone marrow expansion. There was also thickening of the lamina dura, loss of height of the alveolar bone, and distinct radiopaque areas caused by repairs to bone infarctions.^13^ The periapical radiographs showed a coarse trabecular pattern, increased medullary space, bone rarefaction and loss of height of the alveolar bone, thereby minimizing the bone manifestations of the disease in a significant manner. These signs should alert clinicians to the possibility that a patient has sickle cell anemia.^14^ The case reported here is unique, because the patient visited the clinic because of severe jaw pain and not because of carious teeth or dental pain and thus the importance of thorough clinical examination.

## CONCLUSION

Sickle cell anemia presents with variable clinical manifestations, and different degrees of severity that depend on the stage at which this disease is found, the patient’s age, number of hospitalizations, need for blood transfusions and need for continuous drug use, among others. It is important that all clinicians are aware of the physiopathology and oral manifestations of sickle cell anemia and that dental surgeons should carefully obtain the patient’s clinical history and information about particular features so that they can plan any dental treatment such that it is appropriate to the patient’s limitations and needs.
